# Comparability of four clinical laboratory measurement methods for GGT and commutability of candidate reference materials

**DOI:** 10.1002/jcla.23557

**Published:** 2020-09-11

**Authors:** Rui Zhang, Qingtao Wang

**Affiliations:** ^1^ Department of Clinical Laboratory Beijing Chao‐yang Hospital Capital Medical University Beijing China; ^2^ Beijing Center for Clinical Laboratories Beijing China

**Keywords:** commutability, comparability, gamma‐glutamyl transferase, reference method

## Abstract

**Background:**

This study was conducted to evaluate the progress in the standardization of the gamma‐glutamyl transferase (GGT) to achieve metrological traceability of routine in vitro diagnosis (IVD) medical devices.

**Methods:**

We collected 25 single fresh frozen serum samples for GGT analysis. Candidate reference materials (RMs), calibrators, internal quality controls (IQC), and external quality assessment (EQA) materials from the National Center for Clinical Laboratory (NCCL), Beijing Center for Clinical Laboratory (BCCL), and College of American Pathologists (CAP) were randomly added to these serum samples. A total of 42 samples were examined using IFCC reference method and four different IVD medical devices to perform the comparability and commutability study.

**Results:**

The four IVD medical devices achieved trueness assessment within the measurement range. Linear analysis showed the agreement of Siemens ADVIA 2400, Hitachi 7600‐020/BioSino, Beckman AU 5800, and Roche Cobas 501 with the reference method. These assay pairs were comparable at the medical decision levels. The GGT in‐house candidate RMs, and Beckmann and Roche calibrators were all within the limits of the 95% prediction intervals, the commutability of BioSino calibrators was indeterminate, and some internal and external quality controls were not commutable for comparisons of certain IVD medical devices vs the reference method.

**Conclusions:**

By comparing with the reference method, we found that performance of GGT conventional measurement systems to be traceable to the higher order references was improved. The commutable materials for calibration and trueness controls of routine methods were significant to promote the standardization of GGT analysis.

## Graphical abstract

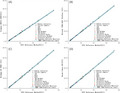

## 1 INTRODUCTION

Gamma‐glutamyl transferase (GGT) belongs to a group of peptidases that catalyze the transfer of amino acids from one peptide to another. GGT is present in all cells of the body except the muscles, and GGT in the serum originates mainly in the hepatobiliary system. GGT is increased significantly in the presence of intrahepatic or posterior biliary obstruction. It is more sensitive than alkaline phosphatase for detecting obstructive jaundice, cholangitis, and cholecystitis, as it increases earlier and persists for longer. Additionally, recent studies showed that serum GGT plays an important role in the monitoring and prognosis of metabolic syndrome and cardiovascular and cerebrovascular diseases^1^, ^2^, ^3^, ^4^ and is a marker of oxidative stress.^5^, ^6^

The detection of serum GGT is one of the most commonly used tests in clinical laboratories. Methods for detecting serum GGT include (a) a reference method recommended by the International Federation of Clinical Chemistry and Laboratory Medicine (IFCC)^7^ which shows accurate measurement results, but the operation is tedious, time‐consuming, costly, and cannot be automated, making it unsuitable for routine detection of clinical patients samples, and (b) conventional measurement systems with simple operation, low‐cost, automation, and wide use. However, the results of conventional measurement systems are often inconsistent; Xia Cet al^8^ showed deviation was as high as 14.2% compared to IFCC reference procedure and coefficients of variation between systems as high to 11.6%. Additionally, some systems are not consistent in the level of medical decision‐making.^9^ Reference methods are rarely used as a basis for comparison in these studies. The purpose of this study was to compare the reference method for GGT recommended by the IFCC with the four conventional measurement systems to evaluate the accuracy and reliability of the measurement results according to EP09‐A3.

Commutable substances can be used to detect and monitor differences between the results of different laboratories in the EQA Plan or Performance Verification (PT). This requires an understanding of the commutability of the substances for EQA or PT. To this end, we assessed the commutability of CAP samples, EQA substances from NCCL and BCCL, and enzymatic in‐house materials developed by the reference laboratory of Chaoyang Hospital to obtain experimental data for the PT program. The conventional measurement system consists of calibrators, quality control products, test reagents, and instrument. There is a metrological traceability from the routine result to the manufacturer's calibrator or even to a reference material and thus SI units. Because of the importance of the commutability of calibrators,^10^, ^11^, ^12^ studies conducted nationally and internationally have demonstrated that applying commutable calibrators can reduce differences in the test results between systems.^8^, ^9^ Therefore, it is necessary to understand the commutability of calibrators used in mainstream methods in China. IQC products are used to monitor the inter‐day stability of the laboratory's internal IVD medical device and evaluate the precision of the method. However, when differences are observed between IQC products and human blood samples, the coefficient of variation does not reflect the actual detection performance of the instrument,^13^ which may affect the detections of the patients. Therefore, in this study, 25 fresh serum samples from patients were used to assess the commutability of the above reference materials among the four commonly IVD medical devices to provide necessary information for relevant decision‐making departments.

## 2 MATERIALS AND METHODS

### 2.1 Reagents and instruments

According to the IFCC reference measurement for GGT, the reagents of the highest purity included *N*‐glycylglycine, (C_4_H_8_N_2_O_3_), L‐γ‐glutamyl‐3‐carboxy‐4‐nitroanilide, monoammonium salt, monohydrate (C_12_H_12_N_3_O_7_·NH_4_·H_2_O), sodium hydroxide solution (NaOH), and sodium chloride (NaCl), all of which were from Sigma. The instruments included a Hitachi U‐3900 UV‐Vis spectrophotometer; a Sartorius pH indicator and LA120S analytical balance; Siemens ADVIA 2400 (matching original reagent, Lot: 354400); Hitachi 7600‐020 (BioSino reagent, Lot: 150821); Beckman AU 5800 (matching original reagent, Lot: AUZ3410); and Roche Cobas 501 (matching original reagent, Lot: 616197).

### 2.2 Ethics statement

The study involved the use of leftover patient serum samples. The leftover patient samples were all de‐identified during the collection. The use of patient samples in the present study has been reviewed by the Ethics Committee of Beijing Chaoyang Hospital. Detailed patient information was not needed, and the data were analyzed anonymously; therefore, participants did not provide written informed consent.

### 2.3 Prepared materials

#### 2.3.1 Calibrators (one level)

BioSino: (Lot: 150085); Beckman Coulter AU5800: (Lot: 0118); and Roche CFAS calibrators (Lot: 176 155‐01).

#### 2.3.2 Controls (2 or 3 levels).

Two‐level EQA materials (lot: 201611 and 201612) were from NCCL; two‐level EQA materials (marked 2016 L1 and L2) were from BCCL; three‐level EQA materials (marked 6, 7, and 10) were from CAP 2015 C‐B program; two‐levels IQC products (lot: 883UN and 649UE) were from Randox company; and two‐level IQC products (lot: 0035 and 0036) were from Beckman company.

The calibrators and quality controls (except from CAP) are provided in the form of a freeze‐dried powder and must be re‐dissolved. Before use, reagent grade laboratory water was added to a bottle based on specification requirement. Each sample was incubated for 30 minutes at 20 ~ 25°C. The bottles were mixed gently until the samples were completely dissolved.

### 2.4 In‐house candidate reference materials

The in‐house candidate RMs for GGT were from the reference laboratory in Beijing Chaoyang hospital (3 levels). These materials were from patient leftover sera which were pooled, mixed thoroughly, sterile‐filtered to 0.20 µm and aliquoted 0.8 mL sera into 1‐mL cryovials and stored under −80°C until use. Three levels of frozen serum RMs were assigned values according to the IFCC reference method for GGT measurement by candidate reference laboratories. One new aliquot of each level was tested four replicates a day for three successive days. Results were expressed as “target value ± uncertainty”. The homogeneity and stability of the candidate RMs were evaluated according to ISO Guide 35.^14^ Ten vials of each concentration were analyzed in triplicate to determine their homogeneity, which were analyzed using one‐way ANOVA. For the stability assessment, linear regression analysis was used. The candidate RMs for GGT were homogeneous and stable for at least 3 days as stored at 4°C and 12 months as stored at −80°C. The in‐house prepared materials as the candidate reference materials met the characterization of the secondary reference materials. We will further apply for the secondary reference materials certification to the National Standard Substance Committee.

### 2.5 Serum samples

Twenty‐five low, medium, and high single fresh frozen serum samples for GGT without hemolysis, lipemia, and icteric were tested and found to be negative for HIV1 + 2 antibodies. These samples were obtained from 2 to 8°C refrigerator in the Department of Laboratory Medicine at the Beijing Chao‐Yang Hospital within 48 hours. The concentrations were 8‐868 U/L. Each sample had a volume of at least 5 mL after mixing evenly, and the samples were divided into five parts and stored at −80°C; the commutability may not be influenced when they were thawed and measured.^15^ The single serum samples were homogeneous and stable for at least 3 days as stored at 4°C and showed stability after 3 months of storage at −20°C.^16^, ^17^

### 2.6 IFCC reference measurement method for GGT

Our laboratory of Beijing Chaoyang hospital has been entered the IFCC RELA EQA program and get acceptable results every year since 2008. We met all management and technical requirements and are preparing the relevant work to apply for the accreditation of the ISO/IEC 17025:2005^18^ and ISO 15195:2003.^19^ The primary reference method was performed strictly according to the IFCC publications.^20^, ^21^ N‐Glycylglycine was weighed 2.73 g (206.3 mmol/L) and dissolved in 100 mL water to prepare the reaction solution by the adjustment of PH. L‐γ‐Glutamyl‐3‐carboxy‐4‐nitroanilide, monoammonium salt, and monohydrate were weighed 0.229 g (33.00 mmol/L) and dissolved in 20 mL water to prepare the start reagent solution. A 2.000 mL reaction solution was added into the cuvette and equilibrated to 37°C, and then, a 0.250 mL sample as a quality control was added (equilibrated close to 37°C). After mixing thoroughly and incubating the sample for 3 minutes, the temperature of the solution in the cuvette reached 37°C. Finally, 0.500 mL start reagent solution was added, mixed thoroughly, and incubated for 60 seconds, after which absorbance was monitored for 3 minutes. Dynamic modeling software was used to analyze the results. The IFCC External Quality Assessment Scheme for Reference Laboratories in Laboratory Medicine (RELA 2014) was used, and samples A and B were used as quality controls, which were required to be within the uncertainty range of the laboratory mean of the RELA 2014 (limit of equivalence). Each sample of prepared materials was tested twice by the IFCC reference method for GGT.

### 2.7 Routine measure systems

Forty‐two samples were tested three times on four types of IVD medical devices including the Siemens ADVIA 2400 (original reagent lot: 354400), Hitachi 7600 (BioSino reagent lot: 150821), Beckman AU5800 (original reagent lot: AUZ3410), and Roche Cobas C501 (original reagent lot: 616197) after internal quality control tests were passed. The IVD devices were operated according to the instructions for use. All the producers stated that their procedures were traceable to the IFCC reference procedure. All the IVD medical devices had been well maintained. The trueness of the routine systems was investigated by the bias compared with target values of candidate RMs, and the means of the replicate measurements were used to calculate the CV.

### 2.8 Comparison and commutability study

According to EP9A‐3,^22^ 25 fresh serum samples were simultaneously evaluated by the reference method and routine measurement systems. All samples were measured twice by the IFCC reference method and three times by four routine IVD medical devices. The mean values were calculated. The results from the 25 fresh serum samples were analyzed by Deming regression analysis. A predicted bias $\hat{B}$ ^22^ (95% confidence interval) at the two medical decision levels Xc (60, 150 U/L) within the allowable bias range (based on moderate biological variation, 11.1%)^23^ indicates good agreement between the routine method and reference method; otherwise, the methods are not comparable.

The commutability experiment for GGT was designed according to the CLSI EP‐14A‐3 guideline.^24^ Prepared materials (calibrators, quality control materials, homemade candidate RMs) together with 25 patient samples were measured for GGT using the four routine IVD medical devices and IFCC reference method. The measurement results were analyzed in a pairwise manner, and Deming regression and 95% prediction intervals were calculated between each routine IVD medical device and IFCC reference method. The commutability of these prepared materials between the reference method and four different analyzed systems was estimated. Prepared materials with measurement results inside the prediction intervals were considered as commutable.

## 3 RESULTS

### 3.1 Trueness assessment of the conventional measurement system

The mean values and expanded uncertainties of the results for GGT candidate RMs at three levels were (174.9 ± 3.7 U/L), (108.3 ± 2.3 U/L), and (32.4 ± 0.8 U/L), respectively. The relative bias of measurements taken from the four routine IVD medical devices compared with the target values were −4.34% to 1.4% (Siemens), −15.1% to −6.2% (Hitachi/BioSino), −9.6% to −1.8% (Beckman), and −10.8% to −2.3% (Roche) (Table 1). Siemens achieved the optimum performance goal (bias, 5.5%). Beckman and Roche met the desirable performance goal (bias, 11.1%). Hitachi/BioSino achieved the minimum performance goal (bias, 16.6%).

**Table 1:** Mean, variance, and bias of GGT activity measurements (U/L) of candidate RMs from four IVD medical devices

RMs	IFCC	Siemens			Hitachi/BioSino			Beckman			Roche		
	Target value	Mean	CV (%)	Bias (%)	Mean	CV (%)	Bias (%)	Mean	CV (%)	Bias (%)	Mean	CV (%)	Bias (%)
L‐1^a^	174.9	177.3	1.4	1.4	162.8	0.1	−6.9	171.8	0.4	−1.8	170.8	0.5	−2.3
L‐2	108.3	109.0	0.9	0.64	101.6	0.5	−6.2	103.8	0.8	−4.2	101	0.6	−6.7
L‐3	32.4	31.0	3.2	−4.34	27.5	0.6	−15.1	29.3	0.3	−9.6	28.9	1.7	−10.8

^a^ The level of candidate RMs.

### 3.2 Comparability of IVD medical devices for GGT

**Figure 1:**
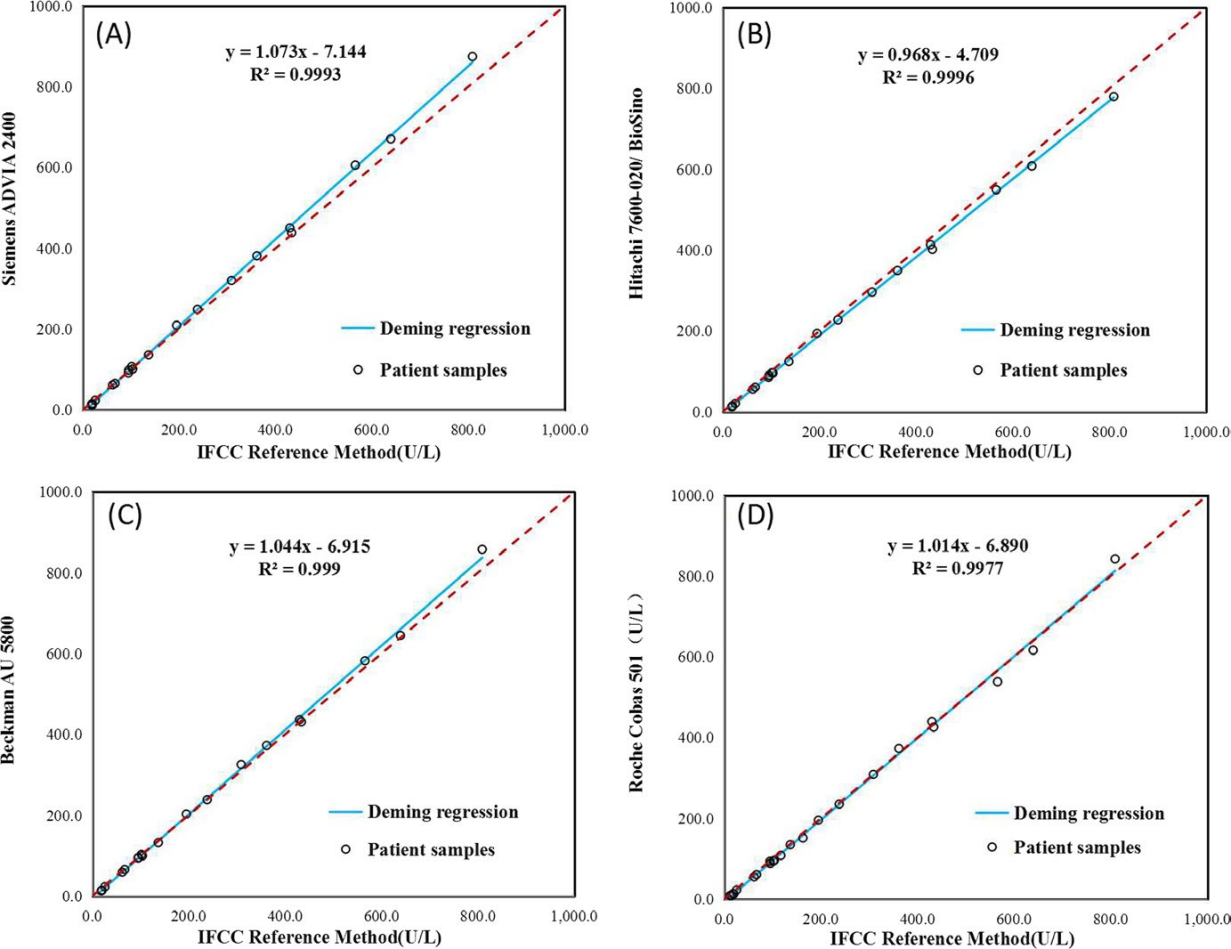
Deming regression and agreement analysis between four IVD medical devices (A‐D) and reference method for GGT. The dashed lines are the equality lines (y = x)

The 25 single fresh frozen serum samples were analyzed on 4 IVD medical devices and IFCC reference method for the comparison experiments. The linear relationships between X (IFCC reference method) and Y (routine methods) are shown in Figure 1 and Table 2. Compared to the reference method, there was a negative bias (intercept is −7.144 to −4.709) between the four IVD medical devices and reference method for GGT. The residual standard deviations (Sy.x) for denotes Siemens ADVIA 2400, Hitachi 7600‐020/BioSino, Beckman AU 5800, and Roche c501 were 6.7, 5.2, 7.3, and 10.5, respectively. The bias between four IVD medical devices and the reference method at the levels of 60 and 150 U/L were shown in Table 3. Compared to the moderate allowable deviation (11.1%) derived from biological variability,^23^ four measurement procedures were acceptable at the levels of 150 U/L medical decision. The 95% confidence limits of Hitachi/BioSino, Beckman, and Roche were exceeded the 11.1% bias acceptability criteria at the 60 U/L medical decision points.

**Table 2:** Deming regression analysis of IVD medical devices

IVD medical devices	Slope(95% CI)^a^	Intercept (95% CI)^a^	*R* ^2^
Siemens	1.073 (1.046‐1.100)	−7.144 (−10.594 to −3.695)	.9993
Hitachi/BioSino	0.968 (0.957‐0.978)	−4.709 (−6.151 to −3.267)	.9996
Beckman	1.044 (1.006‐1.082)	−6.915 (−11.705 to −2.125)	.9990
Roche	1.014 (0.954‐1.073)	−6.890 (−14.067—0.287)	.9977

^a^ Deming slopes and intercepts are expressed as means and 95% confidence intervals (CIs).

**Table 3:** Calculation of whether or not the methods meet the acceptable bias requirements

Xc^a^ (U/L)	Predicted bias $\hat{B}$_c_ (95% CI)^b^ (%)				Acceptable bias (%)
	Siemens	Hitachi/BioSino	Beckman	Roche	
60	−4.62 (−8.34 to −0.89)	−11.05 (−13.23 to −8.87)	−7.15 (−11.86 to −2.45)	−10.10 (−16.60 to −3.50)	11.1
150	2.53 (1.09 to 3.96)	‐6.34 (−7.43 to −5.25)	0.24 (−1.69 to 1.22)	−3.2 (−5.20 to −1.20)	11.1

^a^ Clinical decision level of GGT.

^b^ Deming predicted biases are expressed as means and 95% confidence intervals (CIs) at concentration Xc.

### 3.3 Commutability of prepared materials for GGT measurement

**Figure 2:**
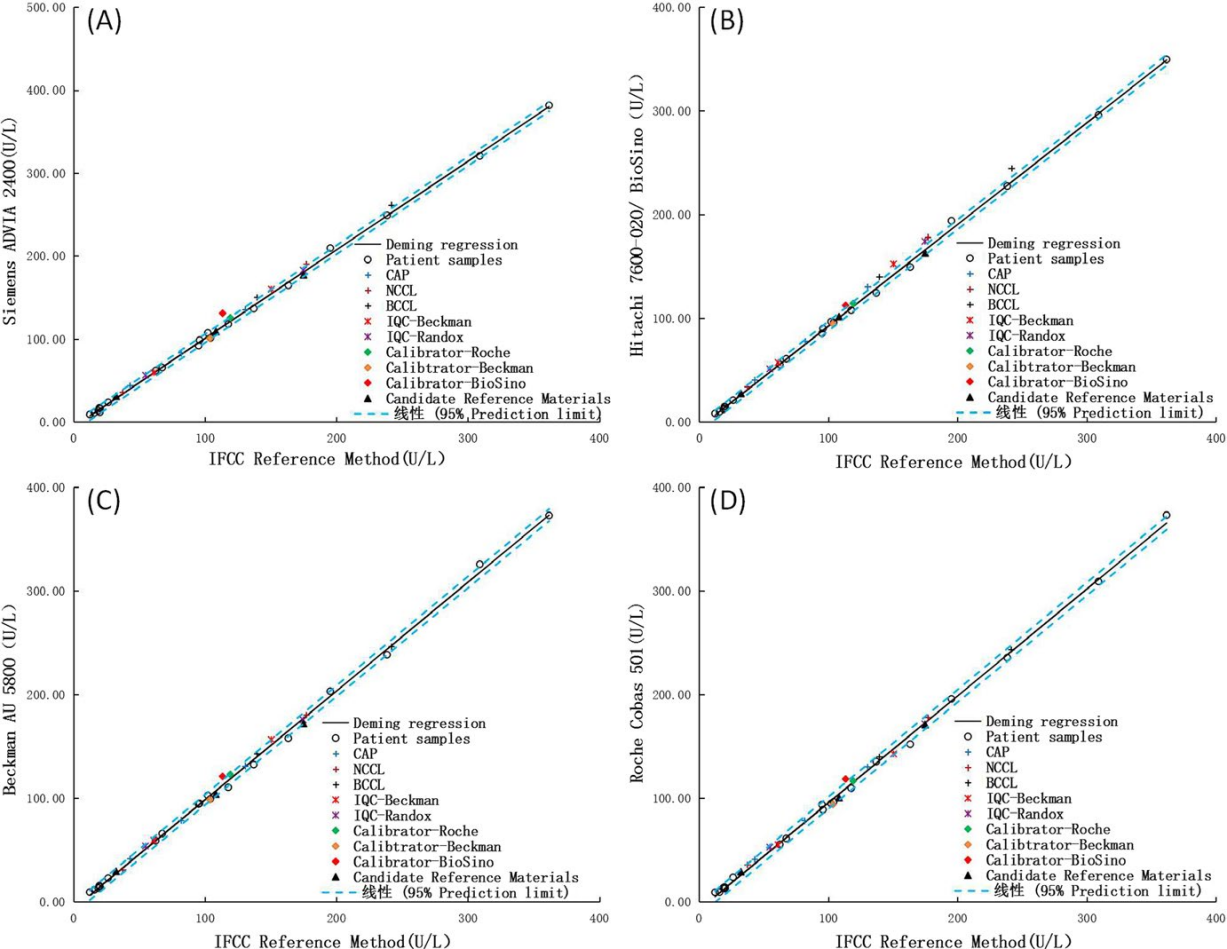
Commutability of reference materials for GGT between IFCC reference method and IVD medical devices (A‐D). The solid lines are the regression lines, and dashed lines are the limits of the 95% prediction intervals. Cross represents NCCL, BCCL, and CAP external quality assessment substances, asterisk represents internal quality control products, diamond represents calibrators, and triangle represents homemade candidate reference materials

Figure 2 Siemens ADVIA 2400‐Roche c501 shows regression line diagrams of 25 serum samples determined by the reference method and four routine methods. The measurement results of the reference method (X) and evaluated method (Y) were analyzed in a pairwise manner by Deming regression. GGT candidate RMs, Beckmann, and Roche calibrators showed good commutability for four comparisons of all IVD medical devices vs the reference method. CAP samples, and Beckman and Randox IQC materials also showed good commutability except Hitachi/BioSino. EQA substances from NCCL and BCCL only were commutable for two comparisons of Beckman and Roche device vs the reference method. BioSino calibrator was noncommutable for Siemens device vs the reference method and the commutability was indeterminate in other devices (see Figure 2 and Table 4).

**Table 4:** Commutability assessment of test materials for four routine methods vs the IFCC reference method according to two different approaches

Test Materials	Method			
	Siemens	BioSino	Beckman	Roche
CAP	1^a^	0^c^	1	1
NCCL	2^b^	0	1	1
BCCL	0	0	1	1
IQC—Beckman	1	0	1	1
IQC—Randox	1	0	1	1
Calibrator—Roche	1	1	1	1
Calibrator—Beckman	1	1	1	1
Calibrator—BioSino	0	2	2	2
Candidate RMs	1	1	1	1

^a^ Commutable.

^b^ Indeterminate.

^c^ Noncommutable.

## 4 DISCUSSION

Obtaining accurate and reliable examination results by clinical laboratories is the goal of laboratory medicine. To achieve accurate test results, testing methods must be standardized to ensure the metrological traceability of the test results. Currently, biochemical reagent manufacturers in China are increasingly considering metrological traceability. We obtained comparable information between the GGT assays in this study, which was significant for the standardization of enzyme.

The CLSI EP09‐A3 is an internationally recognized guideline for comparing different methods.^22^ In this study, Deming regression was used as a method to estimate slope and intercept parameters from a measurement procedure comparison experiment with allowance for both measurement procedures to have imprecision. The measurement error for each measurement procedure is taken into account in the estimation procedure using Deming regression analysis. Table 3 showed deviation of GGT at clinical decision level. The allowable deviation derived from biological variability was divided into low, moderate, and high grades. Jackknife approach provided in the CLSI EP09‐A3 was implemented to calculate Deming slope, intercept, and bias at medical decision points.^22^

Clinical routine methods and reference methods for GGT are based on the same methodological principle of using L‐gamma‐glutamyl‐3‐carboxyl‐4‐nitroaniline as the substrate and diglyceride as the glutamyl receptor. Under catalysis by GGT, a glutamyl group was transferred to the DG molecule and yellow 2‐nitro‐5‐aminobenzoic acid was released. The absorbance increased at 410 nm, and rate of increase in the absorbance was proportional to GGT activity. However, different calibrators and different reagent formulations from different manufacturers may give variable results. Our study revealed negative bias in each system and the intercept for the reference method was −7.144 to −4.709 (Table 2). Additionally, we found that the detection wavelength of Roche Cobas 501 is 415 nm, which differs from other approaches.

EP14‐A3 is an international guideline for evaluating matrix effects or commutability and requires at least 20 representative native patient samples as standards for comparison.^23^ We used 25 native patient samples at low, medium, and high activities, and the data were processed using the commutability assessment method described by the guidelines. Notably, we labeled all samples (serum panel and reference materials) uniformly, and detection staff was blinded to the sample identity. Thus, the detection process was equivalent to a blinded method. We found that CAP samples and EQA substances from NCCL and BCCL lacked commutability among some systems. The reasonability of the evaluation can be ensured only by grouping based on IVD medical devices. The lack of commutability of calibrators may be one reason why the results are not comparable. In this study, we found Beckmann and Roche calibrators showed good commutability between all IVD medical devices and the reference method. The results also showed the Beckmann and Roche systems had reasonable performance goal in the trueness and comparability assessment (Tables 1 and 3).

The GGT in‐house candidate reference materials (3 levels) developed by the human serum pools prepared from patient samples are commutable, which can be further used to certificate national secondary standard substances in the future. The reference materials are useful for external quality assessment and validation of the routine testing systems, as well as for improving the accuracy and consistency of GGT results by different manufacturers.

A major limitation of the study is that all measurements of each method were performed in a single measurement system under specific measuring conditions. The routine methods were performed in four different clinical laboratories, and the reference methods were performed by the reference laboratory. These measuring conditions were assumed to be representative. Therefore, the future measurements should be further performed with the same methods to generalize the research conclusions.

## AUTHOR CONTRIBUTIONS

All the authors have accepted responsibility for the entire content of this submitted article and approved submission.
